# Is There a Persistent Dysfunction of Neurovascular Coupling in Migraine?

**DOI:** 10.1155/2015/574186

**Published:** 2015-02-01

**Authors:** Andrej Fabjan, Marjan Zaletel, Bojana Žvan

**Affiliations:** ^1^Institute of Physiology, Medical Faculty, University of Ljubljana, Zaloška Cesta 4, 1000 Ljubljana, Slovenia; ^2^Department of Vascular Neurology, University Clinical Centre, Zaloška Cesta 2, 1000 Ljubljana, Slovenia

## Abstract

Changes in cerebral blood flow are one of the main features of migraine attack and have inspired the vascular theory of migraine. This traditional view has been reshaped with recent experimental data, which gave rise to the neural theory of migraine. In this review, we speculate that there might be an important link between the two theories, that is, the dysfunction of neurovascular coupling.

## 1. Introduction

Migraine has been traditionally viewed as a vascular disorder [1], but more recent discoveries have led to neural theory of headache origin [2]. The latter postulates that migraine headache is caused by an inappropriate activation of trigeminovascular system, that is, pain sensitive innervation of dural, arachnoid, and pial vessels as well as large intracranial vessels by nociceptive fibers originating in trigeminal ganglion (TG) and travelling mainly through ophthalmic and to a much lesser extent through maxillary and mandibular divisions of trigeminal nerve [3]. Efferent fibers of trigeminal ganglion pseudounipolar neurons converge with upper cervical (C1 and C2) dorsal root nerve fibers in trigeminocervical complex (TCC) [3]. Peripheral nerve endings of TG neurons contain potent vasodilatory neuropeptides such as substance P, calcitonin gene-related peptide, neurokinin A, and pituitary adenylate-cyclase activating peptide, which can be released through an axon reflex and provide efferent potential in pathophysiologic setting such as subarachnoid hemorrhage [4]. On the other hand, stimulation of TG induces an ipsilateral decrease of peripheral resistance in the territory of carotid artery and an increase in facial temperature through trigemino-parasympathetic reflex due to efferent projections of second order neurons of TCC to superior salivatory nucleus [3]. Additionally, TCC makes direct ascending connections with areas of the brainstem, including the ventrolateral periaqueductal grey (PAG), raphe nuclei (RN), and locus coeruleus (LC), which also modulate its nociceptive traffic [5, 6]. Somatosensory and visceral nociceptive information from the head is relayed through TCC to hypothalamus and thalamic nuclei via the trigeminohypothalamic and trigeminothalamic tracts, respectively [6]. The thalamus represents the gateway to central processing of nociceptive information in the so-called “pain matrix,” which includes primary and secondary somatosensory areas, anterior cingulate cortex, and prefrontal cortex [6].

It is believed that activation of the trigeminovascular system follows aberrant cortical activity, such as cortical spreading depression (CSD), epileptic discharge, or following an external electromagnetic stimulation, which stimulate trigeminal nociceptive meningeal nerve endings [3]. Brains of patients with migraine are prone to aberrant cortical activity, as they appear to be hyperresponsive, and have deranged interictal metabolic homeostasis and consequently lower threshold for generating CSD [4]. CSD is thought to activate perivascular trigeminal nerves by way of released potassium, hydrogen protons, nitric oxide (NO), arachidonic acid (AA), and adenosine 5′-triphosphate (ATP) [7]. It induces opening of neuronal Pannexin1 (Panx1) mega-channels and the release of proinflammatory mediators such as high-mobility group box 1 (HMGB1) from neurons, which initiates a parenchymal inflammatory response, leading to sustained release of inflammatory mediators from* glia limitans* and, hence, prolonged trigeminal stimulation [8]. Pial trigeminal afferents are affected primarily, since they are the nearest to* glia limitans*, where concentrations of inflammatory substances are highest and less influenced by continuous cerebrospinal fluid flow, whereas dural afferents are either directly activated or stimulated through an axon reflex-like mechanism of trigeminal axon collateral branches [9].

Brains of migraineurs most likely possess an additional precipitating factor for migraine pain, that is, the dysfunctional modulation of TCC's nociceptive traffic by diencephalic and brainstem areas like hypothalamus, thalamus, PAG, nucleus raphe magnus, and LC [6]. It has been suggested that dysfunctional brainstem modulation of second order neuronal activity in TCC may lead to nociception without a peripheral cause, therefore representing the “migraine generator” [5]. Brainstem dysfunction in patients with migraine has been associated with a more extensive central nervous system imbalance, also referred to as the “migraine state,” responsible for diverse clinical (pre-, peri-, and postictal) features and patterns of widespread brain activity seen in functional neuroimaging studies [10]. Abnormal brain metabolism is a feature of the “migraine state,” as well as abnormal coupling of microvascular blood flow to metabolic needs of brain tissue, that is, the neurovascular coupling (NVC) [11]. Various mechanisms have been proposed, like altered function of astrocytes [10] and impaired subcortical modulation of NVC in migraine [12], since dysfunctional brainstem structures like LC and RN are also implicated in regulation of cortical microvascular tone [13]. Additionally, blood-brain barrier (BBB) permeability is increased after CSD, due to activation and upregulation of matrix metalloproteases [14]. In principle, disruption of BBB renders microvascular wall more susceptible to changes in cortical extracellular milieu, which might have an influence on NVC that could outlast a migraine attack since upregulation of metalloproteases can last for up to 48 hours after CSD [14].

NVC is a mechanism that serves to couple microvascular blood flow to the metabolic needs of surrounding brain tissue, that is, neurons and glia [15]. It is a mechanism distinct from cerebral autoregulation, which prevents noxious variations in cerebral blood volume with oscillations of systemic blood pressure, and will not be discussed further in this paper. Studies have shown that NVC is dependent on the intact function of several cell types, collectively called the neurovascular unit (NVU) [16]. NVU describes the close spatial and functional relationship between neurons, astrocytes, and microvessel wall cells (pericytes, smooth muscle, and endothelial cells) [17]. It has been determined that brain blood flow is most closely coupled to synaptic transmission generating local field potentials and less so to emerging action potentials generating neuronal spiking activity [18]. NVC is a feed-forward mechanism involving binding of released synaptic factors such as glutamate and ATP to receptors on local neurons and astrocytes and unfolding cascades of reactions leading to increased concentrations of potassium and AA metabolites at vascular smooth muscle cells [19]. The emerging response of vessels (dilation or constriction) is dependent on local factors such as oxygen and lactate concentration [20] and is apparently modulated by activity of subcortical noradrenergic, cholinergic, and serotoninergic projections [13]. Vascular endothelium is important in regulating the response and is also a key structure in propagating the dilation upstream to larger vessels [21].

The study of NVC in humans usually employs indirect techniques due to invasiveness of direct approaches [18]. In order to obtain an index of NVC a measure of both regional neural activity and regional cerebral blood flow (CBF) should be assessed [22]. However, far from ideal, majority of human studies in migraine employ only parameters of CBF.

## 2. Neurovascular Coupling between Migraine Attacks

Several studies using single-photon emission computed tomography (SPECT) or Xenon-133 blood flow technique interictally have described abnormalities of CBF in patients with migraine for the most part as hypoperfusion of various cortical areas or asymmetry of cortical blood flow [23–33], although in some of the patients, areas of hyperperfusion were also found [25, 27]. It is interesting that in some of these studies areas of interictal hypoperfusion were more often detected in patients experiencing aura during an attack [27, 32, 34] or in patients suffering from chronic and/or severe migraine [23, 28]. On the other hand, earlier studies with Xenon-133 technique have not detected abnormalities of interictal CBF; however, the method had poorer resolution and was insensitive to regional CBF changes within the thin cortical layer [35, 36]. Nevertheless, the detection of interictal CBF disturbances in migraine both with and without aura led some authors to conclude that both entities might share the same pathophysiological mechanism with cerebrovascular dysregulation [25, 30, 34]. A caveat of such conclusion is that due to method's poor temporal and spatial resolution and lack of correlation of abnormal CBF patterns with neurological, neurophysiological, or neuropsychological deficits in patients with migraine interictally, such finding might be a cause as well as a consequence of migraine [26, 28].

Studies with positron emission tomography (PET) have assessed glucose metabolism as well as regional CBF in patients with migraine interictally [37–41]. The two studies by Denuelle et al. have found relative bilateral hypoperfusion in occipital cortex during an attack compared to the interictal state in migraine with aura and increased luminously stimulated occipital blood flow in patients with migraine during headache and after pain relief with sumatriptan [38, 39]. However, these studies lacked the comparison to healthy controls, whereas another study by the same group using the same methodology found increased luminously stimulated occipital blood flow in migraine patients interictally with and without concomitant painful stimulus compared to healthy controls [37]. This observation could be due to cortical hyperexcitability in patients with migraine as the authors of the study have postulated [37]. However, the lack of concomitant electrophysiological measure of neural activity allows for an alternative interpretation of results in favor of concomitant dysfunction of NVC in patients with migraine, which could be most pronounced under painful conditions when the difference between the two groups of subjects in the study was least obvious [37]. Additionally, Kassab et al. have shown a significant increase in resting glucose uptake in posterior supratentorial and infratentorial white matter in patients with migraine during interictal period, suggesting a metabolically hyperactive, dysfunctional subcortical tissue in its unprovoked state [40].

Functional magnetic resonance imaging using blood oxygenation level dependent signal (BOLD fMRI) has become firmly established as the method of choice in neuroscience to assess areas of altered neural activation and/or functional connectivity between brain regions. However, although it is widely accepted that BOLD signal is indirectly driven by neural activity, the method directly images the proportion of deoxygenated hemoglobin dependent on the combination of regional CBF, blood volume, and tissue oxygen consumption rate, which can in certain conditions and specific brain areas be dissociated from electrical activity of neurons [42]. Using different visual stimuli, studies have mainly shown increased BOLD signal or decreased threshold of activation in visual cortex in patients with migraine interictally compared to healthy subjects [43–45]. On the other hand, a concomitant electrophysiological and fMRI study by Bramanti et al. has demonstrated increased interictal cortical excitability with decreased occipital BOLD signal activation compared to healthy subjects implicating dysfunctional NVC [46]. However, as authors have pointed out, a possibility exists that visual stimulation protocol in that study initiated CSD and consequent period of cortical hypoactivation in patients with aura [46]. Again, similarly to PET, BOLD fMRI studies implicate an increased responsiveness of visual cortex in migraine but do not clarify the nature of the phenomenon, namely, is it of neural and/or vascular origin [42]. For this reason, fMRI studies on pain processing in migrainous brain could have an inherent drawback of nonlinear regional BOLD signal increases/decreases with change in neural activity, since pain is known to activate brainstem structures [47], implicated also in modulation of NVC [13], that could be dysfunctional in migraine [6].

Transcranial doppler (TCD) ultrasound offers great temporal resolution and has been used extensively in studies on CBF in patients with migraine interictally [48–59]. A major limitation of the technique's use in studies on NVC is that it is a blind method (no data on vessel diameter) that measures changes in blood flow velocity in large arteries at the base of the scull, that is, anatomically distant from microvasculature, with no information on neural activity [60]. Alternatively, it can be combined with electroencephalography to estimate an index of NVC [22] or with BOLD fMRI to covisualize the extent and intensity of activated cortical tissue [61]. The majority of TCD studies on visually evoked blood flow velocity response in the posterior cerebral artery (VEFR) have found increased response in patients with migraine compared to healthy subjects interictally [48, 49, 53, 56, 58]. However, two more recent studies found no difference [61, 62]. The study by Griebe et al. is interesting not only for the methodological aspect, but also for yielding conflicting results since increases in cortical BOLD signal response to optokinetic stimulus were not accompanied by increases in VEFR, although a slower restitution of evoked hemodynamic response was observed in patients with migraine [61]. Additionally, significant asymmetry of VEFR was found by Wolf et al. in migraine with aura, which did not, however, statistically correlate with lateralization of neurological symptoms during an attack, although VEFR was higher on the headache side in majority of patients [63]. Nevertheless, appropriate side selection of region of interest is important in studies on NVC in migraine as pathophysiological mechanism might be asymmetrical and therefore more or less lateralized [64]. Our study has shown that cold pressor test has no effect on VEFR in patients with migraine, whereas in healthy subjects it causes an increase, suggesting dysfunctional NVC in migraine under tonic pain condition [65]. We have speculated this to be due to dysfunction of subcortical modulatory projections involved in the regulation of microvascular tone.

TCD studies using various vasoactive agents like altered CO_2_ partial pressure, l-arginine, and acetazolamide have demonstrated altered vasomotor reserve in migraine interictally [51, 52, 54, 57, 66–77]. The most consistent finding in these studies was increased vascular reactivity to hypocapnia and decreased reactivity to a vasodilatory agent (i.e., acetazolamide or l-arginine) in patients with migraine, implicating decreased tone of cerebral vessels in migraine and/or dysfunction of cerebrovascular endothelium [54, 67, 68, 73, 75]. On the other hand, hypercapnia induced heterogeneous cerebrovascular responses with either hyperreactivity, hyporeactivity or no difference in reactivity [51, 52, 57, 69–72, 74, 77]. Studies on patients with migraine with aura show more abnormalities than in patients without aura [67, 68, 71, 73, 76]. In addition, posterior circulation might be particularly prone to cerebrovascular abnormalities in migraine [52, 54, 71]. Altered cerebrovascular vasomotor reserve suggests dysfunction of vascular elements of NVU, although this assumption should be viewed with caution since animal studies have demonstrated the action of CO_2_ to be most pronounced on pial arterioles, which are upstream from NVU [78].

In general, using different investigative techniques in patients with migraine studies have demonstrated high incidence of alterations in CBF interictally, which are probably related to ongoing pathophysiological process between attacks [25, 30, 37, 39, 41, 51]. However, the isolated finding of altered CBF in the absence of concomitant data on neural activity hampers a valid conclusion on possible dysfunction of NVC in patients with migraine. On the other hand, electrophysiological studies have provided conflicting results that do not elucidate whether there is increased or decreased cortical activity interictally [79]. Furthermore, studies that have evaluated changes in CBF together with changes in cortical activity are pointing to an altered rate of NVC in migraine interictally [46, 58, 61].

## 3. Neurovascular Coupling during Migraine Attack

During migraine with aura there is a characteristic reduction in CBF of posterior cerebral regions which coincides with the symptoms of aura as was first shown by intracarotid Xenon-133 method studies [80, 81]. In these studies, oligemia was sometimes preceded by focal hyperemia and had the tendency to slowly propagate anteriorly with the rate of 2 mm per minute [81, 82]. Oligemia could reach critical, even lower than ischemic values of cerebral perfusion, albeit rarely [82]. Headache ensued during oligemia and continued during hyperemia, which usually outlasted headache [80].

Subsequent PET studies have found decreased cerebral perfusion in migraine up to 24 hours after the onset of headache [83–85]. In a PET study of nine subjects with spontaneous migraine without aura, global CBF and volume were found to be lower by 9.9% and 5.2%, respectively [84]. In this study, trend toward increased oxygen metabolism and extraction in the headache phase was found in five of nine patients despite reduced flow suggestive of uncoupling of flow and metabolism; however, no significant change in oxygen metabolism could be found in other patients, making the observation statistically insignificant [84]. A PET study of alcohol-triggered migraine attacks (with and without aura) showed a parallel decrease in flow and metabolism (23.1% and 22.5%, resp.) with unchanged oxygen consumption rate in the primary visual cortex [83]. A report by Woods et al. described bilateral profound (up to 40%) decrease in blood flow starting in visual associative regions and spreading continuously across the cortical surface at a relatively constant rate, spanning different vascular territories in a female patient with migraine without aura who developed an attack being scanned in PET device for other reasons [85]. This observation again raises the possibility of a similar pathophysiological mechanism in migraine with and without aura. Other PET studies failed to demonstrate perfusion abnormalities in patients with migraine without aura during an attack; however the focus of their investigation was the brainstem [64, 86].

A perfusion-weighted fMRI study has demonstrated that migraine with visual aura causes moderate decreases in occipital perfusion, which corresponded neuroanatomically with visual aura symptoms and side of headache [87]. The maximal reduction of perfusion was above ischemic threshold and resolution of visual symptoms was followed by improvement of perfusion (not vice versa), contradicting a hypothesis that visual aura is ischemic in nature [87]. Further study confirmed these findings but failed to demonstrate the same changes in migraine without aura or in other brain regions outside occipital lobes during visual aura [88]. In addition, the same study found perfusion deficit in one patient experiencing migraine both with and without aura only during the aura, which indicates that decremental blood flow changes are more specific for migraine with aura [88]. A study on BOLD response during migraine visual aura revealed marked suppression to light modulation in visual cortex that has spread with a rate corresponding to progression of hemifield scintillations described by a subject [89]. In this study, time-dependent BOLD activity changes in the region of interest implicated a sequence of cortical gray matter blood flow changes beginning with hyperemia, followed by hypoperfusion with attenuated response to visual activation and subsequent return of resting blood flow to baseline with recovery of stimulus driven activation [89]. Similarly, albeit somewhat less time- and space-defined changes were described in an earlier BOLD fMRI study [90]. Spatial and temporal distributions of these changes were similar to features of CSD observed in animals, corroborating the hypothesis that CSD is the pathophysiological substrate of migraine aura [91–93].

Several TCD studies have shown decreased blood flow velocities in middle cerebral artery during migraine attack [34, 94–96]. This probably reflects decreased CBF as the diameter of large cerebral arteries remains constant during moderate perturbations of arterial blood pressure and CO_2_ levels [97]. Speculation on NVC based on these data is difficult, as correlation with data on cortical activity during migraine attack is needed. Previous studies have shown attenuated somatosensory evoked potentials during sensory aura, which returned to normal during the headache phase [98]. Conversely, during migraine with persistent visual aura, sustained visual cortex hyperexcitability was demonstrated with visual evoked magnetic field measurements [99]. During ictal and peri-ictal periods a vast body of evidence points to normalization of cortical excitability in migraine both with and without aura [100]. Therefore, changes in CBF during those periods can be viewed as a consequence of dysfunctional NVC.

## 4. Putative Mechanisms of Dysfunctional Neurovascular Coupling in Migraine

It appears that migraine attacks occur in connection with exacerbations of preexisting changes of neural activity, CBF, and metabolism during the interictal period [33, 101]. In migraine with aura, CSD is likely a central event initiating ictal disturbance in neural activity and CBF [102]. A study measuring multiple parameters of neural and vascular response in mice has shown that the propagating CSD wave initiates two large desaturations of cortical hemoglobin, the first during the CSD wave and the second, after a brief recovery, in its aftermath, which may reach levels observed during middle cerebral artery occlusion and points to mismatch between tissue metabolic demands and vascular response [103]. The response of microvessels during CSD wave in this study was vasoconstriction, as opposed to proposed vasodilation in initial hyperemia in humans [89]. However, vasoconstrictive response with CSD was observed also in humans with subarachnoid hemorrhage [104]. Furthermore, animal studies have shown tissue hypoxia even with vasodilation of microvessels suggesting impaired NVC that can last several hours after CSD [105]. Interestingly, Brennan et al. demonstrated vasomotor changes to be conducted ahead of the electrophysiological changes of CSD wavefront in mice and rats, implicating CSD to be composed of distinct vascular and neural components [106].

After CSD, the vascular reactivity is markedly impaired in response to CO_2_ partial pressure, transcallosal fiber stimulation, vasoactive substances, and basal forebrain stimulation [105, 107–109]. Interestingly, blood pressure autoregulation of CBF remains intact [108]. Nitric oxide (NO) synthase inhibition has no effect on CBF increases or oligemia in normal rats [110]. On the other hand, increase in NO levels due to l-arginine administration prevents development of prolonged oligemia after CSD and speeds the recovery of reduced vascular reactivity but has no influence on the marked rise of CBF during CSD [110]. The effect of increased NO levels might be due to vasodilatory actions of increased vascular smooth muscle cyclic guanosine monophosphate or impeded vasoconstriction due to inhibition of AA conversion to potent vasoconstrictor 20-hydroxyeicosatetraenoic acid (20-HETE) [111]. This mechanism is relevant because CSD causes a significant increase in tissue AA through glutamate receptor induced increases in neuronal and astrocytic cytosolic calcium and activation of phospholipases A and C [112]. Consequently, there is an increase of 20-HETE levels, which correlates with the persistent decrease in CBF after CSD [113]. However, blocking of 20-HETE production does not prevent impaired NVC after CSD [113]. Therefore, CBF reduction and impaired NVC are probably accounted for not only by increased 20-HETE production but also by other pathophysiological mechanisms initiated by decreased NO availability as a result of CSD [113].

Vasoconstrictive (or inverse) NVC might be an important mechanism in CBF abnormalities that could lead to clinical consequences in patients with migraine and as such an important target for therapeutic interventions. A study on transgenic mice bearing familial hemiplegic migraine type 1 mutation of neuronal voltage-gated calcium channels has demonstrated enhanced susceptibility to anoxic and peri-infarct depolarization as well as larger perfusion defects during ischemic depolarization attributed to inverse NVC [114]. Therefore, mechanistically similar mutations, for example, mutation of glial glutamate transporter EAAT2 associated with common migraine [115], might render susceptible patients with migraine more prone to develop CSD after mild ischemic vascular events, such as microembolisms, and more sensitive to stroke with mild ischemia [114]. Indeed, patients with migraine, especially migraine with aura, have higher prevalence of clinically silent infarct-like lesions in the territory of posterior circulation compared to normal controls [116]. Additionally, migraine with aura has been associated with a twofold increase in stroke risk [117], which appears to rise with increasing migraine attack frequency [118]. Migraine prophylactic therapy might be especially important in these patients [119].

The association of migraine and stroke also implies that cerebrovascular endothelium might be impaired in migraine [120]. Some even suggest that migraine is a systemic vasculopathy [121]. Level of endothelial progenitor cells, a biological marker of vascular dysfunction, was reduced in patients with migraine in several studies [122, 123]. However, endothelium dependent vasodilatation of systemic circulation was unimpaired in migraine [124]. On the other hand, endothelin-1, a potent vasoconstrictor, was capable of inducing CSD even more readily than potassium in vivo in rats [125], whereas in patients with migraine, its levels were elevated during the attack free period [126]. It remains to be determined if markers of disturbed endothelial function are a consequence of a general systemic response in migraine or an essential part of the disease's pathophysiological process in cerebral vessels.

Several studies have implicated dorsal pontine area in the region of LC to be activated during spontaneous and induced migraine attacks and even in the premonitory phase of an induced attack [86, 127, 128]. LC provides the majority of brain's noradrenergic neurons with widespread projections throughout the cerebral cortex that end on pyramidal cells, GABA interneurons, astrocytes and microvessels [129–132]. LC stimulation increases noradrenaline release in the cortex and promotes both cortical neuronal and astrocytic activity [133–135]. On the other hand, several in vivo animal studies have demonstrated reduced intraparenchymal vessel diameter or decrease in global cortical blood flow with LC stimulation [136–139], which is counterintuitive with respect to concomitant increase in neural activity and in disagreement with a recent study by Toussay et al. [140]. A study by Bekar et al. has enabled better understanding of the issue by demonstrating that the noradrenaline-mediated global decrease in vessel diameter improves synchronization of both temporal and spatial characteristics of the sensory stimulation-mediated hyperemia and surround blood volume decrease locally [141]. In this regard, the absence of LC's ability to optimize redistribution of blood flow to areas where vasodilatory signals overpower noradrenergically mediated vasoconstriction [142] might contribute to abnormalities of basal and evoked cerebral blood flow observed in patients with migraine due to exaggerated hyperemic or oligemic regional response, with the latter possibly subsidizing an inverse NVC or even initiating CSD. In this respect, it is interesting that in cats the reduction of regional CBF with stimulation of LC was most pronounced in the occipital cortex [137], where spreading oligemia during migraine aura has been demonstrated [82]. However, topical administration of noradrenergic agonists and antagonist (noradrenaline, clonidine, and propranolol) has attenuated CSD in rats with no effect on regional CBF [143], and clonidine infusion had no influence on posterior CBF as assessed by PET in healthy human volunteers [144], making the role of LC dysfunction in initiating a migraine attack less clear.

Cortical serotonin levels are decreased between migraine attacks, whereas during an attack brain serotonin synthesis increases [145, 146]. Dysregulation of the brainstem serotonin system has been proposed in patients with migraine during a pain-free interval [147]. Pharmacological degeneration of serotonergic neurons in dorsal raphe nucleus (DRN) significantly increases velocity of SD and extends the width of the depolarization wave in rats [148]. Additionally, serotonin depletion likely augments hyperemic response after CSD in rat cerebral cortex due to increased NO production [149]. Serotoninergic projections are important in regulating cerebral microvascular tone [150]; however DRN stimulation produces a heterogeneous response in different cortical areas [151]. Since DRN is deeply interconnected with other brainstem areas implicated in migraine attack (e.g., LC) [152], the importance of DRN dysfunction as a primary phenomenon in altered NVC in migraine needs to be further proven.

## 5. Conclusion

Changes in CBF and cerebrovascular reactivity in different phases of migraine as measured by different investigative methods are profound and point to an important role of cerebral vasculature in the pathophysiology of the disease. Impairment of NVC that has been demonstrated in animal models is most likely a key feature of migraine headache in humans. This is in line with the theory of “migraine state,” a complex derangement of cerebral homeostasis that eventually leads to migraine headache [10]. In this regard, the impairment of NVC could be viewed as common ground for the vascular and neural theory of migraine.

## 6. Future Directions

It remains to be established whether changes in cerebrovascular reactivity in migraine are disease's cause or consequence. According to the theory of “migraine state,” episodic or persistent change in cortical extracellular environment could be expected in patients with migraine. It would be interesting to measure different extracellular cortical substances implicated in the pathophysiology of migraine headache in vivo in selected cases, for instance, with the use of microdialysis (e.g., in migraine patients undergoing brain/cranial surgery for other reasons).

Studies on cerebrovascular reactivity on patients with chronic migraine are needed. More profound abnormalities are expected as chronic migraine is regarded as a clinical worsening. Comparison should be made to patients with episodic migraine during and outside of an attack. The study of pathophysiological mechanisms that eventually leads to the chronicity of the disorder is important in the development of specific acute and prophylactic therapy.

Additionally, due to a probable nonlinear cortical NVC in migraine [42], results of functional BOLD fMRI in migraine, which assert that the magnitude of the BOLD response is in linear correlation with local neural activity, should be compared to alternative imaging methods more reliant on either neural metabolism or local cerebral blood flow (e.g., 18-fluoro-deoxyglucose PET or arterial spin labeling fMRI studies).
